# Complementarities of occupations and language skills of immigrants in Europe

**DOI:** 10.1007/s10663-024-09636-w

**Published:** 2024-11-21

**Authors:** Peter Tóth, Matej Vitáloš

**Affiliations:** 1 Department of Economic Policy, Faculty of Economics and Finance, University of Economics in Bratislava, Bratislava, Slovakia; 2 Supreme Audit Office of the Slovak Republic, Bratislava, Slovakia; 3 Research Department, National Bank of Slovakia, Bratislava, Slovakia

**Keywords:** Migration, Returns to language skills, Occupational sorting, Language-skill complementarities, J15, J31, J61

## Abstract

We study the returns to language skills of immigrants using the European Adult Education Survey (2016). We estimate a standard income equation augmented by self-reported proficiency levels in the host country’s language and in English. Contrary to earlier literature, we find that the inclusion of English skills of immigrants increases the estimated returns to proficiency in the local language. Next, considering heterogeneous effects across occupations, we find significantly positive returns to language proficiency only for medium-skilled occupations. Among those, blue-collar jobs reward fluency in both the local language and English. Whereas in white-collar jobs, only the knowledge of English yields significantly higher income. These estimates are consistent with occupational sorting of immigrants and suggest that there are complementarities between proficiency in languages and job skills for some occupations. Following earlier literature, we also corrected the potential endogeneity bias in host-country language skills using instrumental variable methods. Our findings could be relevant for immigration policies in Europe.

## Introduction

A large body of literature documents language skills as important determinants of immigrants' labor market outcomes in host countries. The knowledge of the host country's language, as well as other foreign languages, increase the probability of employment and have a positive effect on income. This is not surprising, as language skills satisfy all of the basic characteristics of human capital: they are embodied in the person; they are productive in the labor market and/or in consumption; and they are created at a sacrifice of time and out-of-pocket resources (Chiswick and Miller 1995). As Berman et al. (2003) conclude, language may well be the most important public good in a society, it is non-rival in use and provides network externalities. To the extent that language provides externalities, estimated private returns may understate the social returns to language training. Consequently, there may be under-investment in language skills in competitive equilibrium, especially in case of immigrants. Therefore, supporting language classes for immigrants not only speed up their economic assimilation but may also provide a general social benefit through improved communication.

**Fig. 1:**
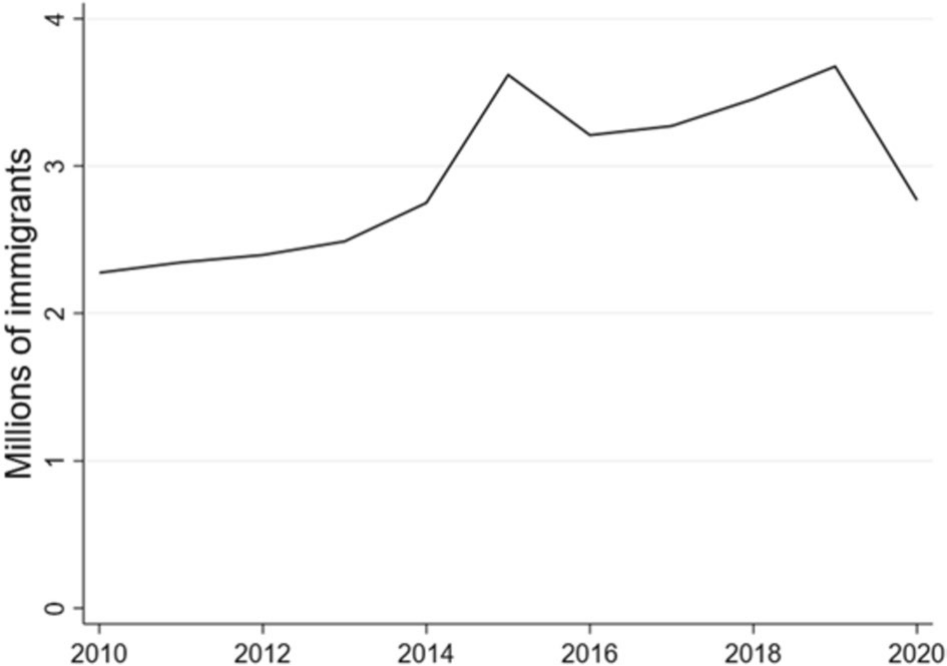
Annual migration flows to Europe Source: Authors’ calculations based on data from the Eurostat database. Note: EU27 countries plus Norway and Switzerland. Immigrants born in the reporting country are excluded

Figure 1 shows that immigration in Europe peaked during 2015–2019, and compared to the pre-2015 period, annual flows are expected to remain high in the post-covid years (Acostamadiedo et al. 2020; Grieveson et al. 2021). Based on trends of the recent years and the high expected social return to the provision of accessible and high quality language training for immigrants, studying this topic is increasingly important for the European economy.

We contribute to the literature on the returns to language skills of immigrants with new evidence from a multi-country dataset of 29 European countries. Using Eurostat's Adult Education Survey (2016), we exploit information on self-assessed proficiency levels in multiple languages the respondent may report. As one of the novelties of our paper, we consider immigrants' proficiency in both the host country's language and in English, which was only studied in Lang and Siniver (2009). In contrast to the conclusions of the above authors, however, we find that including English proficiency in the income equation does affect the estimated returns to fluency in the host country's language. Namely, the estimated returns increase as a result. This may suggest the correlation of English proficiency with general unobserved skills of immigrants and, if ignored, may lead to biased estimates. Further, we show that differentiating the levels of language proficiency matters for estimating income premia, however the common practice in prior research was to use binary indicators. Our estimates suggest that only an almost-native level of fluency in the host country's language yields significantly higher income to immigrants. Whereas in case of English, any additional level of proficiency has a positive effect on income.

As a further contribution, we consider heterogeneous returns to language skills across standard occupation categories. For the subsamples of low-skilled, medium-skilled blue-collar, medium-skilled white-collar and high-skilled occupations we find significantly positive returns only for medium-skilled jobs. While the English skills of immigrants are an asset in both blue- and white-collar jobs, fluency in the local language is rewarded only in blue-collar jobs. Our estimates add new evidence to previous findings in the literature suggesting that language proficiency tends to complement some types of human capital and jobs in a more enhancing way than others, which likely leads to the occupational sorting of immigrants (see e.g. Berman et al. 2003; Boyd and Cao 2009; Dávila and Mora 2000; McManus et al. 1983).

Following the existing literature, we also attempt to correct for the potential endogeneity of language skills in the income equation by instrumental variable (IV) methods. First, we use linguistic proximity of the local language to the immigrant's native language and a proxy for age at immigration as instruments for language skills (following Isphording 2013; Clarke and Isphording 2017; Ghio et al. 2023). To explore further IV options, we additionally consider heteroskedasticity-based instruments following Lewbel (2012).  The first-stage diagnostic tests suggest that all the instruments are relevant and are correlated with the endogenous explanatory variable of host-country language skills. However, the IV methods yield estimates that are either not statistically significant or are very close to the baseline of ordinary least squares (OLS).

## Literature review

In one of the first papers on this topic, McManus et al. (1983) study the effect of English language skills on earnings of Hispanic men. The authors argue that the potential inability to communicate in modal languages is associated with lower earnings. They explain this point using a simplified framework, where communication skills of employees may be valuable to the firm in several aspects, such as the verbal interaction between producers and customers, interpersonal communication within the production process, as well as certain type of communication between labor and production capital. As regards empirical evidence, the authors use data on earnings from 1975 to explore the role of English language proficiency in the assimilation of Hispanic men in the US labor market. Their estimates reveal that once English language skills are taken into account, wage differentials associated with Hispanic ethnicity, US nativity, schooling abroad, and time in the United States are no longer statistically significant. The authors interpret this result as the mediation effect of language skills on the mentioned factors. The paper also provides evidence that that negative effects of host-country language deficiency rise with occupational skill level. Tainer (1988) uses the same data and shows that language proficiency affects the earnings of various ethnic groups differently. Generally, Hispanics and Asians have larger language effects than Europeans, but language proficiency improves earnings of all foreign-born men.

Chiswick and Miller (1995) use ordinary least squares (OLS), instrumental variables (IV), and sample selection techniques to study the determinants of dominant language fluency and its effects on earnings of immigrants in Australia, United States, Canada, and Israel. The analysis is based on the standard human capital earnings function modified for immigrant adjustment (Chiswick 1978), where the natural logarithm of earnings is the function of education, length of labor market experience, duration in the destination, marital status, citizenship, size of dwelling, country of birth and a measure of dominant-language fluency. The authors find that language fluency is associated with higher earnings, at least in case of the OLS estimates. However, if the endogeneity of language fluency is corrected using IV^1^ methods, the results are mixed. In case of Australia and Canada, dominant language fluency turns statistically insignificant, and the coefficient is even negative for Australia. While for the United States and Israel, the IV estimates are positive, statistically significant, and about three times higher than the OLS results. In the next stage of the analysis, the authors use the estimates of the labor market benefits of language fluency to compute the rate of return on investment. As one would expect, investment in language fluency appears to be the most profitable for those immigrants who are not fluent in the dominant language.

Similarly, examining the effect of English language skills on wages among individuals who immigrated to the United States as children, Bleakley and Chin (2004) find evidence of substantial downward bias in the OLS estimate compared to an IV approach.^2^ Because much of the effect of English language skills appears to be mediated by years of schooling, the authors argue that adult English-language classes may be insufficient to help these immigrants' wages to converge to those of natives. Instead, programs aimed at junior-high-school-aged and high-school-aged children may be more effective. This study uses microdata from the 1990 U.S. Census. Further, Schmid (2023) estimates the effect of proficiency in the local language on employment outcomes of African asylum seekers in Switzerland, who were randomly assigned to French- or German-speaking cantons. Exploiting exogenous variation in the placement of immigrants, the author finds that language proficiency more than doubles the employment level in the first five years after arrival.

Boyd and Cao (2009) focus on the Canadian labor market. In addition to finding a positive relationship between levels of language proficiency and earnings, they highlight the mediating role of occupations. In other words, the allocating impact of language proficiency accounts for about one-fifth of the wage gap between the highest and lowest proficiency levels. Moreover, their quantile regression analysis reveals that the earnings gaps between those with high and low levels of host-country language proficiency are greatest for immigrants in well-paying jobs.

Unlike most papers, Berman et al. (2003) study immigration to a non-English speaking country, specifically from the former Soviet Union to Israel in the early 1990s. They find that Hebrew fluency had almost no effect on wage growth in the low-skilled occupations. Moreover, gas station attendants and construction workers show no evidence of wage convergence. In contrast, computer technicians and software engineers benefit from considerable wage convergence, but most of the convergence can be explained by increasing Hebrew fluency among workers in these occupations. However, one must be cautious in extending these results to all low-skilled and high-skilled occupations. According to the authors, it is more accurate to conclude that language complements certain job skills more than others.

Using German micro data from years 2005–2009, Heizmann et al. (2017) estimate the effect of a higher concentration of immigrants in certain occupations on wages of natives and immigrants, also differentiating between blue-collar, white-collar, and highly qualified occupations. The results indicate that the concentration of immigrants is associated with wage devaluation on account of skill quality sorting. In case of white-collar jobs, however, the authors find further wage devaluation attributed to ethnic or cultural differences. At the same time, returns to proficiency in German language for immigrants are not statistically significant.

Budría and Swedberg (2015) explore the impact of Spanish language proficiency on immigrant earnings in Spain. Their results suggest that the earnings gains from host-country language proficiency in Spain are significant, but lower than in other countries. In addition, acquiring Spanish language proficiency is a profitable investment even for less educated immigrants. Nevertheless, there are profound differences in the earnings premium between immigrants with diverse levels of educational attainment, as immigrants with less than upper secondary education gain substantially lower returns from Spanish proficiency than highly educated immigrants. The authors report higher returns in case of IV estimates, compared to standard OLS.^3^

Also focusing on immigrants in Spain, Isphording (2013) examines the returns to foreign language skills, while proficiency in Spanish is not considered. The results highlight the key role of foreign language skills as a part of the human capital portfolio of immigrants. The estimates indicate significant wage premia for proficiency in English, French and German. The largest estimated returns in case of English can be explained by its general importance as a lingua franca in international trade and in Internet and communication technologies. Further, the author finds occupational choice to be an important mediating channel through which foreign language proficiency affects earnings. As regards sensitivity of the results to estimation methods, Isphording (2013) uses a linguistic dissimilarity index as an instrument for language skills and reports three to four times higher estimates under the IV setup, compared to OLS. This suggests the possible endogeneity of language skills with regards to earnings.

Other studies suggesting a positive impact of English language skills of non-natives in countries, where English is not an official language include e.g., Lang and Siniver (2009) for Israel and Toomet (2011) for the Baltic states. As regards interference between English and local language skills, only the former study performs such analysis and concludes that returns to proficiency in the local language do not change significantly if the knowledge of English is also considered.

Papers on foreign language skills and labor market outcomes of all residents, i.e., without the native vs. immigrant distinction, include for example Di Paolo and Tansel (2015, 2019) both using Turkish data and Gazzola and Mazzacani (2019) based on a sample from Germany, Italy, and Spain, all utilizing data from the Adult Education Survey. Another study by Fabo et al. (2017) adds similar evidence from Central European job vacancy data. All the mentioned papers report positive effects of foreign language proficiency on labor market outcomes.

## Methodology and data

### AES data and model specification

The Adult Education Survey (AES) is coordinated by Eurostat and is one of the main data sources for EU lifelong learning statistics. Although its primary focus is on the participation of individuals aged 25–64 in education and training, it also contains self-reported language skills, information on income and other important characteristics. A question about the country of birth permits the analysis of the circumstances of immigrants. So far three waves of the survey have been implemented. The AES 2007 was a pilot survey conducted in 26 EU Member States (Ireland and Luxembourg did not participate) plus Norway, Switzerland, and Turkey. The AES 2011 was conducted in 27 EU Member States (Croatia did not participate) plus Norway, Switzerland, Turkey, and Serbia. The third and latest survey (AES 2016) was conducted in all 28 EU Member States plus Norway, Switzerland, Turkey, Serbia, Albania, Bosnia and Herzegovina and the Former Yugoslav Republic of Macedonia. The 2022 wave of the survey was not yet available at the time of preparing this paper.

Table 1 lists the number of AES 2016 respondents by place of birth for each country. Regrettably, the data lacks detailed information on the countries of origin of immigrants, other than the classification into EU and non-EU countries. To get further insights into the composition of the sample of immigrants, we examined the list of most frequently spoken mother tongues. The languages exhibit a wide range of diversity, with most of them originating from Western Europe, Eastern Europe, the Balkans, and Russia. Among the non-European languages, Arabic and Turkish are the most prevalent. In the French sample, for example, the latter two account for as much as 30% of immigrants, while the corresponding figures for the remaining countries range from 5 to 20%.

**Table 1:** AES 2016 respondents by place of birth

Country	Native-born residents	Immigrants from another EU country	Immigrants from a non-EU country
AT	4626	434	560
BA	6149	112	129
BE	4322	376	394
BG	6491	7	32
CH	5694	1568	997
CY	2402	271	391
CZ	11,944	216	112
DE	6656	335	747
DK	3018	145	266
EE	3328	40	465
EL	5008	103	338
ES	20,690	569	1754
FI	2815	65	95
FR	12,844	429	1,680
HR	2602	42	292
HU	8125	113	59
IE	3956	603	303
IT	14,473	143	228
LT	3295	14	136
LU	1887	1,633	467
LV	5071	61	664
MK	7417	34	150
MT	1780	70	113
NL	2773	73	246
NO	2156	176	217
PL	17,992	29	73
PT	13,022	280	908
RO	15,253	1	3
RS	4444	221	327
SE	2407	143	426
SI	4833	0	0
SK	3192	45	8
UK	6257	279	507

Source: Authors’ calculations based on the AES 2016 data

In our analysis, we focus on the following seven Western European countries (WE7 henceforth): Austria, Belgium, France, Germany, Luxembourg, the Netherlands, and Switzerland. We motivate our choice by the facts that these samples have a sufficient share of immigrants (see Table 1), the countries are similar in terms of culture and local languages. Moreover, two of their local languages are common for more countries (German and French). These similarities suggest that the WE7 represent a distinguishable block within Western Europe. To check the sensitivity of our results to reducing the sample, we also consider an extended set of 29 European countries (E29 henceforth). Here we excluded six of the thirty-five participant countries in the AES 2016 survey. Three of these have English as an official language (Ireland, Malta, United Kingdom). In the remaining three excluded countries, some of the key variables for our paper are missing or are not fully consistent with the rest of the countries (Albania, Slovenia, and Turkey).

To determine the role of language in the process of economic assimilation of immigrants in Europe, we estimate the following regression model:

$$ \begin{aligned} hhincome_{i} = &\, \beta_{0} + \beta_{1} hatlevel_{i} + \beta_{2} exp_{i} + \beta_{3} exp_{i}^{2} + \beta_{4} restime_{i} \\ & + \beta_{5} {\text{deg}}\_urb_{i} + \beta_{6} marstadefacto_{i} + \beta_{7} citizen_{i} + \beta_{8} birthplace_{i} \\ & + \beta_{9} country_{i} + \beta_{10} lang_{i} + \beta_{11} eng_{i} + \varepsilon_{i} , \\ \end{aligned} $$

where *hhincome* is the quintile of the equivalized household total net monthly income,^4^*hatlevel* codes the respondent’s highest level of education or training successfully completed, *exp* is the total potential labor market experience, and *restime* measures the respondent’s years of residence in the country of residence. With the following four variables, we control for the degree of urbanization of the area the respondent lives in (*deg_urb*), the respondent’s de facto marital status including a consensual union (*marstadefacto*), his/her citizenship (*citizen*), and whether he/she was born in the EU or outside the EU (*birthplace*). Country dummies are denoted by *country*. Variable *lang* codes host-country language skills, while *eng* captures the individual's knowledge of English.

Our baseline model is similar to that estimated by Chiswick and Miller (1995). However, there are some minor differences. First, the dependent variable is the quintile of the equivalized household total net monthly income as opposed to the midpoints of the income intervals. Second, the education variable codes the highest level of education or training successfully completed rather than years of schooling. Third, due to the anonymization procedure, the microdata do not contain the 2-digit ISO code of the country of birth. The birthplace variable codes only three aggregated groups: country of survey, another EU country, and a non-EU country.

Importantly, the coding of language skills in the AES 2016 is very similar to that used by Chiswick and Miller (1995)—four categories of knowledge of the language of the country of residence.^5^ In addition to these four categories, we create an additional category for those whose mother tongue is the same as the language of the country of residence. We assign the highest language proficiency to this group of immigrants. Table 2 lists the codes and labels of all levels of knowledge of the language of the country of residence used in our analysis.

**Table 2:** Language proficiency levels

Code	Label
1	I only understand and can use a few words and phrases
2	I can understand and use the most common everyday expressions. I use the language in relation to familiar things and situations
3	I can understand the essential of clear language and produce simple text. I can describe experiences and events and communicate fairly fluently
4	I can understand a wide range of demanding texts and use the language flexibly. I master the language almost completely
5	Mother tongue

Source: AES 2016 data

The same categorization is used for English language proficiency, the effect of which we also study. In this case, however, we work with an additional category for those who did not list English as their first or second best-known language (except their native language), nor did they list English as the language they use.

Total potential labor market experience is not observed in the data, so we use a proxy calculated as the year of interview minus the year of completion of the highest level of education or training, which disregards periods of unemployment. Further, for those who have no formal education or below ISCED 1, we calculate the total potential labor market experience as the age of the respondent minus 15 years (hypothetical age of entry of these individuals into the labor market). We make additional adjustments for those who did not answer the question regarding the year of completion of the highest level of education or training. Details of the procedure will be provided upon request.

In the analysis of occupational sorting, we use the International Labor Organization's (ILO) International Standard Classification of Occupations (ISCO). The high-skilled (HS) occupations include managers (ISCO group 1), professionals (group 2) and technicians and associate professionals (group 3). The medium-skilled white-collar (MSWC) category consists of clerical support workers (group 4) and service and sale workers (group 5). Medium-skilled blue-collar (MSBC) occupations include skilled agricultural, forestry and fishery workers (group 6), craft and related trades workers (group 7) and plant and machine operators, and assemblers (group 8). Low-skilled (LS) jobs are elementary occupations in ISCO group 9.

We focus on male immigrants aged 18–64 and disregard women on account of the extra complications derived from potential selectivity bias, as there may be a problem with the non-random participation of women in the labor market (Budría and Swedberg 2015; Casale and Posel 2011). Moreover, this restriction allows the comparability of our results with most other papers that focus on male subsamples (Berman et al. 2003; Chiswick and Miller 1995; McManus et al. 1983; Tainer 1988; Toomet 2011).

Descriptive statistics of the sample and frequencies of categorical values used in the analysis are reported in Tables 3 and 4 below.

**Table 3:** Summary statistics

Variable	Sample	N	Mean	SD	Min	Max
Income quintile	WE7	3700	2.751	1.472	1	5
	E29	7126	2.740	1.453	1	5
Years of residence	WE7	3700	9.081	3.223	1	11
	E29	7126	9.556	2.866	1	11
Potential experience	WE7	3700	22.342	12.808	0	54
	E29	7126	23.130	12.870	0	54
Marital status	WE7	3700	0.722	0.448	0	1
	E29	7126	0.707	0.455	0	1
Citizenship	WE7	3700	0.357	0.479	0	1
	E29	7126	0.437	0.496	0	1
*Language distance*						
To host country	WE7	3510	37.498	25.696	0	64.308
To English	WE7	3510	16.341	14.822	0	62.575

Source: Authors’ calculations based on the AES 2016 data

**Table 4:** Frequency table for categorical variables

	Host country								
	WE7	AT	BE	CH	DE	FR	LU	NL	E29
*Host-country language proficiency*									
1	5.3	7.7	5.8	6.1	4.0	3.0	5.5	8.2	5.2
2	9.9	10.3	8.1	9.1	14.4	8.5	11.1	8.2	8.1
3	17.8	20.4	13.6	23.1	27.3	12.4	14.5	6.1	14.2
4	23.4	28.8	19.8	21.6	33.3	25.8	16.0	28.6	20.1
5	43.6	32.7	52.7	40.1	21.0	50.2	52.9	49.0	52.4
*English proficiency*									
0	12.4	6.8	13.6	3.8	12.1	28.7	8.6	7.1	18.9
1	38.7	44.4	30.2	41.5	48.6	38.3	33.7	18.4	35.8
2	9.1	10.1	11.2	10.4	11.8	9.5	4.3	12.2	9.4
3	15.5	16.3	17.8	19.4	12.9	11.2	14.7	22.4	14.5
4	19.3	19.1	22.9	20.3	10.9	7.7	31.7	25.5	16.8
5	5.1	3.3	4.3	4.6	3.7	4.6	7.0	14.3	4.6
*Country of birth*									
Another EU country	50.0	42.4	48.1	63.5	29.6	20.9	81.6	23.5	39.5
A non-EU country	50.0	57.6	51.9	36.5	70.4	79.1	18.4	76.5	60.5
*Education attainment*									
No formal education	3.6		14.7	1.3		2.6	6.3	9.2	3.4
ISCED 1	8.9	19.1	6.2	1.1	11.5	12.3	8.8	2.0	7.5
ISCED 2	13.6		10.5	18.2	24.1	18.8	7.6	12.2	15.5
ISCED 3	34.0	47.3	18.6	39.4	33.3	36.8	21.9	43.9	38.2
ISCED 4	1.5		1.9		6.0	0.1	3.4		2.2
ISCED 5	5.6	13.4	2.7		1.1	10.6	5.5	1.0	5.4
ISCED 6	12.4	20.2	13.6	14.3	12.6	7.2	10.3	15.3	10.9
ISCED 7	20.5		31.8	25.6	11.2	11.7	36.1	16.3	16.9
*Occupations (ISCO)*									
LS	8.2	11.6	7.4	4.2	18.6	11.8	4.2	9.9	9.9
MSBC	28.8	40.4	21.0	24.3	36.7	36.5	21.6	21.1	32.3
MSWC	15.8	14.2	15.9	17.5	16.3	16.4	13.9	16.9	17.3
HS	47.3	33.7	55.7	53.9	28.5	35.3	60.3	52.1	40.6
*Degree of urbanisation*									
Cities	49.2	47.5	69.8	44.4	46.8	66.2	30.3	68.4	50.5
Towns and suburbs	32.4	31.6	27.5	41.2	44.3	20.7	33.5	14.3	30.9
Rural areas	18.4	20.9	2.7	14.4	8.9	13.1	36.1	17.3	18.6
Observations	3,700	455	258	895	348	832	814	98	7,126

Source: Authors’ calculations based on the AES 2016 data

LS are low-skilled, MSBC are medium-skilled blue-collar, MSWC are medium-skilled white-collar, and HS are high-skilled occupations. WE7 countries are AT, BE, CH, DE, FR, LU and NL. E29 countries are 24 EU members AT, BE, BG, CY, CZ, DE, DK, EE, EL, ES, FI, FR, HR, HU, IT, LT, LU, LV, NL, PL, PT, RO, SE, SK; potential EU members BA, MK, RS; and non-members CH and NO

### Linguistic proximity and age at immigration as instrumental variables

As language proficiency indicators in the data are based on self-reported information, they may suffer from measurement error. Respondents may not be willing or able to correctly evaluate their own language skills, which can lead to attenuation bias and underestimation of coefficients by OLS. Further, an upward bias in the estimated returns to language skills may result from the omission of unobserved skills or self-selection (see Isphording 2013). Additional bias of an ambiguous direction may result from other potential sources of endogeneity between language skills and earnings (see e.g., Chiswick and Miller 1995). The common solution to these estimation issues is the application of instrumental variable methods if appropriate instruments are available.

Following Isphording (2013), we consider linguistic proximity between the host country's official language to the immigrant's native language as instruments for language skills (denoted as IV1 henceforth). Linguistic differences could be a relevant source of exogenous variation that affects success in learning foreign languages. At the same time, the impact of linguistic factors on income of immigrants is likely to materialize mainly via language skills, as an indirect channel. Therefore, the restriction to exclude this variable from the income equation and using it as an instrument for language skills may be valid.

The proximity measures for constructing our first instrument (IV1) were calculated using distance matrices of the Automated Similarity Judgment Program (ASJP, version 2.1) available on the ASJP website,^6^ with a higher value reflecting a higher lexical similarity or proximity, similarly to the approaches of Isphording (2013), Clarke and Isphording (2017), and Ghio et al. (2023). Table 12 in the Appendix lists the manual language codes changes that were necessary to maximize the number of observations, as quite frequently the AES and AJSP coding was different. In cases where broader language groups (e.g., Arabic, Chinese, etc.) was selected by the respondent as his/her native language, we changed it to one of the most common individual language within each group. A special case was, for example, the Serbian language, which was absent from the ASJP database. In the case of countries with more than one official language (e.g., Belgium, Luxembourg, Switzerland), we consider the host-country language in which the immigrant is the most proficient.

As a refined version of the above instrument, Clarke and Isphording (2017) and Ghio et al. (2023) interact linguistic proximity by age at immigration. The latter indicator could be a further relevant source of exogenous variation in language skills for two reasons. First, learning foreign languages at a younger age increases the probability of mastering the language. Second, immigration decisions at a younger than adult age are made by parents and so self-selection based on higher unobserved skills of a young immigrant may be limited. Unfortunately, our dataset censors information on the years of immigrant status from eleven and above. At the same time, most of the sample includes immigrants who had stayed eleven or more years in the host country. Therefore, we are not able to determine our respondents' age at immigration exactly. Given this limitation, we multiply linguistic proximities by a dummy that equals one if years since immigration exceed ten and use this product as the second version of our instruments (IV2 henceforth).

A further limitation that results from taking an IV approach is due to the measurement of language skills on a five-point scale in our data. As identification of the effects of endogenous variables requires at least as many instruments as the number of instrumented variables and we have only two IVs available, we cannot use separate dummies for each level of language skills. So, for the purposes of the IV analysis, we either use a binary indicator of high^7^ vs. low skill levels or we make a cardinality assumption and treat language skills as continuous variables.

### Using heteroscedasticity-based instruments for language skills

For cases when no external instrumental variables are available, Lewbel (2012) suggests constructing instruments based on heteroscedasticity. We include estimates following such an approach for comparison with the standard IV estimates described in the previous subsection. Borrowing the notation of Baum and Lewbel (2019), heteroscedasticity-based instruments (HSIV) are defined as follows. Consider endogenous variables *Y*_*1*_ and *Y*_*2*_ and a vector of exogenous covariates *X*. In the context of our paper, *Y*_*1*_ is income and *Y*_*2*_ are host-country language skills of immigrants. The main parameter of interest is the effect of language skills on income (*γ*) in the first of the two equations below:

$$ Y_{1} = X^{\prime}\beta + Y_{2} \gamma + \varepsilon_{1} $$

$$ Y_{2} = X^{\prime}\alpha + \varepsilon_{2} $$

where error terms $\varepsilon_{1}$ and $\varepsilon_{2}$ may be correlated. In the above setup we are not certain, whether any element of vector *β* is equal to zero, meaning that an instrument may not be available for the estimation of *γ*. The approach of Lewbel (2012) requires three main assumptions to identify *γ*. First, suppose error terms $\varepsilon_{1}$ and $\varepsilon_{2}$ have the following factor structure:

$$\varepsilon_{1} = cU + V_{1}$$

$$\varepsilon_{2} = U + V_{2}$$

where *c* is a constant and *U*, *V*_*1*_ and *V*_*2*_ are unobserved error components that are mutually independent, conditional on *Z*, and *Z* are some or all elements of X excluding the constant term. In our context, U could be thought of as unobserved ability, which affects both income and host-country language skills of immigrants. Unlike *U*, each of the other two unobservable components, *V*_*1*_ and *V*_*2*_, are specific to only one of the endogenous variables. The remaining two identifying assumptions require that *U* is homoscedastic, i.e. *U*^*2*^ is not correlated with Z, and that *V*_*2*_ is heteroscedastic, implying that $\varepsilon_{2}^{2}$ is correlated with *Z*.^8^

Given the above conditions, the HSIV estimator of Lewbel (2012) can be obtained in two steps. First, estimate *α* by means of an OLS regression of *Y*_*2*_ on *X* and express fitted residuals $\hat{\varepsilon }_{2} = Y_{2} - X^{\prime}\hat{\alpha }$. Second, estimate *γ* and *β* by two-stage least-squares of *Y*_*1*_ on *X* and *Y*_*2*_, using X and $\left( {Z - \overline{Z}} \right)\hat{\varepsilon }_{2}$ as instruments, where $\overline{Z}$ is the mean of $Z$.

## Results

### Effect of language skills on the income of immigrants

In the baseline setup we estimate the income equation outlined in Sect. 3 with different versions of language skill indicators and standard control variables. The second column of Table 5 includes local language skills indicated by various levels of proficiency, instead of the binary indicator in column (1). The results suggest that only the two highest levels of proficiency in the local language yield significantly higher income. The third column of Table 5 importantly shows that the inclusion of the English proficiency variable, which is itself statistically significant, also increases the effect of host-country language proficiency. Column 4 shows an increased estimate of the highest level of proficiency in the local language (compared to column 2) once English skills are included. At the same time, all levels of English proficiency yield additional gains in income.

**Table 5:** Returns to language skills of immigrants (OLS estimates)

	(1)	(2)	(3)	(4)	(5)	(6)
Country of origin of immigrants:	All countries				Extra-EU	All countries
Sample of host countries:		WE7			WE7	E29
Years of residence	0.0666*** (0.0157)	0.0616*** (0.0157)	0.0676*** (0.0156)	0.0631*** (0.0156)	0.0743*** (0.0189)	0.0585*** (0.0126)
Born outside the EU	− 0.644*** (0.0950)	− 0.642*** (0.0949)	− 0.607*** (0.0935)	− 0.602*** (0.0933)		− 0.536*** (0.0681)
Host-country language proficiency	0.134*** (0.0364)		0.160*** (0.0350)			
Host-country lang. prof. (level 2)		− 0.0457 (0.141)		− 0.00722 (0.140)	0.0853 (0.153)	− 0.0712 (0.117)
Host-country lang. prof. (level 3)		0.0694 (0.160)		0.101 (0.159)	0.119 (0.180)	− 0.00164 (0.127)
Host-country ﻿language proficiency (level 4)		0.422*** (0.155)		0.440*** (0.151)	0.475*** (0.176)	0.324*** (0.116)
Host-country ﻿language proficiency (level 5)		0.388** (0.157)		0.584*** (0.169)	0.770*** (0.210)	0.455*** (0.128)
English proficiency			0.189*** (0.0341)			
English proficiency (level 1)				0.380** (0.157)	0.459** (0.189)	0.305*** (0.107)
English proficiency (level 2)				0.535*** (0.179)	0.471** (0.231)	0.463*** (0.124)
English proficiency (level 3)				0.758*** (0.156)	0.699*** (0.190)	0.679*** (0.109)
English proficiency (level 4)				0.817*** (0.185)	0.714*** (0.221)	0.763*** (0.122)
English proficiency (level 5)				0.987*** (0.246)	1.373*** (0.270)	0.890*** (0.183)
Socio-demographic controls	Yes	Yes	Yes	Yes	Yes	Yes
Country dummies	Yes	Yes	Yes	Yes	Yes	Yes
R^2^	0.232	0.236	0.257	0.261	0.245	0.247
Observations	3700	3700	3700	3700	1851	7126

Source: Authors’ calculations based on the AES 2016 data. International weights are used

Robust standard errors are reported in parentheses

***p < 0.01, **p < 0.05, *p < 0.1. WE7 countries: AT, BE, CH, DE, FR, LU, NL. E29 countries are 24 EU members AT, BE, BG, CY, CZ, DE, DK, EE, EL, ES, FI, FR, HR, HU, IT, LT, LU, LV, NL, PL, PT, RO, SE, SK; potential EU members BA, MK, RS; and non-members CH and NO. Socio-demographic controls include: education attainment level, part-time dummy, potential experience, degree of urbanisation, marital status, birthplace and citizenship

Restricting the analysis to immigrants from outside the EU, the results in column (5) of Table 5 change somewhat. The stronger estimated language proficiency effects in the fifth column of Table 6 suggest that language skills are more important for this subset of immigrants. Although the estimated coefficients for proficiency levels 1 and 2 are still statistically insignificant, the point estimates increased compared to the full sample. Overall, the positive effect of host-country language skills in the third and the fifth column are due to the two highest levels of language proficiency. The estimates are similar in magnitude for the sub-sample of full-timers (column 6).

**Table 6:** Returns to language skills of immigrants in WE7 countries by occupations (OLS)

	(1)	(2)	(3)	(4)	(5)	(6)	(7)	(8)
	All immigrants				Non-EU immigrants			
	LS	MSBC	MSWC	HS	LS	MSBC	MSWC	HS
Host-country lang. prof. (level 2)	0.0278 (0.275)	0.619** (0.245)	− 0.889* (0.498)	− 0.734 (0.477)	0.182 (0.492)	1.035*** (0.242)	− 0.516 (0.575)	− 1.000 (0.871)
Host-country lang. prof. (level 3)	0.435 (0.344)	0.413* (0.249)	− 0.754 (0.509)	− 0.418 (0.470)	0.881 (0.619)	0.690*** (0.245)	− 0.591 (0.583)	− 0.190 (0.697)
Host-country lang. prof. (level 4)	0.0432 (0.419)	0.559** (0.235)	− 0.456 (0.510)	− 0.343 (0.411)	− 0.00882 (0.566)	0.971*** (0.210)	− 0.548 (0.580)	− 0.309 (0.653)
Host-country lang. prof. (level 5)	0.311 (0.545)	0.607** (0.280)	− 0.578 (0.549)	0.0466 (0.404)	0.153 (0.848)	1.294*** (0.325)	− 0.735 (0.672)	0.0548 (0.670)
English proficiency (level 1)	0.279 (0.507)	0.140 (0.304)	0.100 (0.472)	0.288 (0.358)	− 0.311 (0.800)	0.386 (0.349)	− 0.158 (0.516)	0.501 (0.516)
English proficiency (level 2)	− 0.171 (0.578)	0.235 (0.312)	0.817 (0.578)	0.302 (0.326)	0.277 (0.808)	0.144 (0.387)	0.0889 (0.670)	0.571 (0.413)
English proficiency (level 3)	0.368 (0.624)	0.454 (0.323)	1.025** (0.420)	0.354 (0.321)	0.122 (0.796)	0.385 (0.400)	1.261*** (0.479)	0.335 (0.432)
English proficiency (level 4)	0.138 (0.603)	1.146** (0.502)	1.092** (0.500)	0.322 (0.333)	0.0565 (0.915)	0.754* (0.446)	0.960 (0.610)	0.313 (0.426)
English proficiency (level 5)	0.819 (0.619)	0.0762 (0.533)	0.429 (0.665)	0.688 (0.424)	0.659 (0.764)	0.340 (0.557)	0.160 (0.812)	1.568*** (0.504)
Socio-demographic controls	Yes	Yes	Yes	Yes	Yes	Yes	Yes	Yes
Country dummies	Yes	Yes	Yes	Yes	Yes	Yes	Yes	Yes
R^2^	0.247	0.183	0.399	0.178	0.276	0.238	0.432	0.212
Observations	215	758	416	1245	150	416	213	407

Authors’ calculations based on the AES 2016 data

LS are low-skilled, MSBC are medium-skilled blue-collar, MSWC are medium-skilled white-collar, and HS are high-skilled occupations following the ISCO classification. International weights are used. Robust standard errors are reported in parentheses

***p < 0.01, **p < 0.05, *p < 0.1. WE7 countries: AT, BE, CH, DE, FR, LU, NL. Socio-demographic controls include: education attainment level, part-time dummy, potential experience, degree of urbanisation, marital status, birthplace and citizenship

In the last column of Table 5 we checked the sensitivity of our findings to including 29 European countries in the sample,^9^ which nearly doubled the number of observations available for estimation. The results are similar to the baseline for WE7 countries in column (4) of the same table, except that the estimated returns are somewhat lower, but still statistically significant.

Table 13 in the Appendix shows that the above findings are robust to using the alternative estimation approach of ordered logit. Although we prefer the computationally simpler OLS, one could also consider this nonlinear approach in case of an ordered categorical dependent variable, such as income deciles. The fourth column of this table shows that for a one level increase in host-country language proficiency (or English proficiency), the odds of being in a higher income quintile are approximately 30% higher. The fifth column of the table shows that the two highest levels of host-country language proficiency are associated with a 132% and 173% higher probability of being in a higher income quintile (compared to level 1). Although statistically insignificant, the estimated coefficients for levels 1 and 2 are higher than 1 (as we would expect). This column also shows that higher levels of proficiency in English are associated with a 73–318% higher probability of being in a higher income quintile.

### Occupational sorting and language-skill complementarities

In this section we estimate the income equation for each occupational category separately to check whether more proficient speakers are better paid in certain types of professions. Such evidence would suggest the presence of occupational sorting and complementarities between language skills and specific job types. We follow the ISCO classification of occupations and group them into broader categories, such as low-skilled (LS), medium-skilled blue-collar (MSBC), medium-skilled white-collar (MSWC) and high-skilled (HS) jobs.^10^ Table 6 shows that language skills bring significant returns for immigrants only in medium-skilled occupations. Particularly for MSBC jobs, both the knowledge of the local language and level-4 fluency in English are associated with a positive and significant effect on income. As for MSWC jobs, the knowledge of the local language is negatively correlated with income, although the relationship is not statistically significant. Instead, what seems highly valuable in these clerical, service and sales jobs is a level 3 or 4 fluency in English. The above pattern of estimates indicates specific complementarities between different sets of language skills and job characteristics, which can lead to occupational sorting.

As for related findings in the literature, McManus (1983) and Berman et al. (2003) also show that language proficiency complements certain types of human capital more than others. Dávila and Mora (2000) find that Mexican immigrants in the U.S. with poor English fluency tend to sort into low-skilled jobs. Using data from Germany, Heizmann et al. (2017) report that a higher concentration of immigrants in certain occupations is associated with wage devaluation on account of skill quality sorting, but returns to proficiency in German language for immigrants are not statistically significant. Further, McManus (1983) shows evidence of increasing returns to host-country language skills for higher occupational skill levels, which is partially consistent with our results. Next, Berman et al. (2003) report higher language returns for higher skilled workers. Although the authors included only computer technicians and software engineers as two high-skilled occupations and gas station attendants and construction workers as two low-skilled occupations.

Further, we checked the sensitivity of our occupation-specific estimates to extending the sample to 29 European countries (Table 7). The results are robust only partially, as returns to fluency in the local language are smaller and no longer statistically significant for MSBC occupations. While English proficiency levels 2–4 all bring statistically significant returns for immigrants in both MSBC and MSWC jobs.

**Table 7:** Returns to language skills of immigrants in E29 countries by occupations (OLS)

	(1)	(2)	(3)	(4)	(5)	(6)	(7)	(8)
	All immigrants				Non-EU immigrants			
	LS	MSBC	MSWC	HS	LS	MSBC	MSWC	HS
Host-country lang. prof. (level 2)	0.111 (0.244)	0.236 (0.239)	− 0.296 (0.269)	− 0.280 (0.355)	0.373 (0.408)	0.228 (0.326)	− 0.134 (0.268)	− 1.143** (0.475)
Host-country lang. prof. (level 3)	0.516* (0.302)	0.122 (0.230)	− 0.110 (0.278)	− 0.281 (0.350)	0.887* (0.514)	0.00761 (0.316)	− 0.124 (0.283)	− 0.426 (0.429)
Host-country lang. prof. (level 4)	0.377 (0.338)	0.240 (0.225)	0.251 (0.259)	− 0.0860 (0.286)	0.442 (0.478)	0.342 (0.302)	0.227 (0.263)	− 0.422 (0.384)
Host-country lang. prof. (level 5)	0.473 (0.360)	0.289 (0.249)	0.0848 (0.308)	0.208 (0.282)	0.630 (0.503)	0.574 (0.350)	− 0.107 (0.373)	− 0.0994 (0.395)
English proficiency (level 1)	0.0435 (0.286)	0.149 (0.207)	0.112 (0.282)	0.104 (0.243)	− 0.103 (0.339)	0.360 (0.238)	− 0.160 (0.315)	0.241 (0.351)
English proficiency (level 2)	− 0.291 (0.319)	0.430** (0.214)	0.692* (0.385)	0.289 (0.216)	0.0118 (0.352)	0.528** (0.254)	0.170 (0.475)	0.535* (0.286)
English proficiency (level 3)	0.152 (0.354)	0.502** (0.238)	0.937*** (0.246)	0.352* (0.211)	− 0.119 (0.438)	0.540** (0.272)	0.857*** (0.277)	0.422 (0.290)
English proficiency (level 4)	0.450 (0.579)	0.852** (0.349)	0.759** (0.311)	0.371* (0.209)	0.992 (0.692)	0.516 (0.329)	0.520 (0.363)	0.374 (0.275)
English proficiency (level 5)	0.230 (0.344)	0.104 (0.422)	0.283 (0.442)	0.806*** (0.302)	0.143 (0.393)	0.464 (0.483)	0.0264 (0.524)	1.468*** (0.366)
Socio-demographic controls	Yes	Yes	Yes	Yes	Yes	Yes	Yes	Yes
Country dummies	Yes	Yes	Yes	Yes	Yes	Yes	Yes	Yes
R^2^	0.226	0.160	0.298	0.186	0.220	0.160	0.297	0.203
Observations	497	1,622	870	2,040	369	1,015	568	937

Source: Authors’ calculations based on the AES 2016 data

LS are low-skilled, MSBC are medium-skilled blue-collar, MSWC are medium-skilled white-collar, and HS are high-skilled occupations following the ISCO classification. International weights are used. Robust standard errors are reported in parentheses

***p < 0.01, **p < 0.05, *p < 0.1. E29 countries are 24 EU members AT, BE, BG, CY, CZ, DE, DK, EE, EL, ES, FI, FR, HR, HU, IT, LT, LU, LV, NL, PL, PT, RO, SE, SK; potential EU members BA, MK, RS; and non-members CH and NO. Socio-demographic controls include: education attainment level, part-time dummy, potential experience, degree of urbanisation, marital status, birthplace and citizenship

### Treating the endogeneity of language skills

Table 8 compares OLS and IV results for three sets of instruments and two measures of language skills, where host-country language skills are instrumented and the knowledge of English is treated exogenous. In case of binary language skills (columns 1–4), only the IVHS coefficient estimate on host-country language skills is statistically significant, and is similar in value to the OLS estimate.

**Table 8:** Returns to language skills of immigrants in WE7 countries (OLS vs. IV)

	(1)	(2)	(3)	(4)	(5)	(6)	(7)	(8)
	OLS	IV1	IV2	IVHS	OLS	IV1	IV2	IVHS
Host-country language prof. (Dummy: ≥ level 3)	0.409*** (0.095)	0.719 (0.506)	− 0.882 (1.410)	0.414*** (0.106)				
Host-country language prof. (cardinal measure)					0.189*** (0.037)	0.134** (0.058)	0.016 (0.089)	0.190*** (0.042)
English proficiency (Dummy: ≥ level 3)	0.429*** (0.113)	0.424*** (0.114)	0.448*** (0.117)	0.429*** (0.113)				
English proficiency (cardinal measure)					0.176*** (0.037)	0.170*** (0.037)	0.158*** (0.038)	0.176*** (0.037)
First-stage coefficient of IV		0.091***	0.037***			0.802***	0.584***	
Kleibergen-Paap rank LM underidentification test (χ^2^)		116.73***	11.65***	414.28***		337.55***	174.95***	599.04***
Hausman test χ^2^, OLS vs. IV		1.37	2.78	0.03		6.63	17.80	42.66***
Observations	3510	3510	3510	3510	3510	3510	3510	3510

Source: Authors’ calculations based on the AES 2016 and ASJP data

International weights are used. Robust standard errors are reported in parentheses

***p < 0.01, **p < 0.05, *p < 0.1. WE7 countries: AT, BE, CH, DE, FR, LU, NL. Socio-demographic controls and country dummies were included. In columns IV1, IV2 and IVHS, host-country language proficiency is instrumented by: language proximity (IV1), language proximity interacted with age at immigration (IV2), and the heteroscedasticity-based instruments of Lewbel (2012) (IVHS), respectively

Point estimates based on the other two IVs are statistically insignificant and shift up or down compared to OLS, depending on the version of instruments used. The coefficients on English skills are positive and statistically significant in all four specifications. Looking at standard diagnostics of the relevance of instruments, the first-stage coefficients of the instruments are statistically significantly positive (columns 2 and 3). This is the expected sign, as a higher proximity of languages and a longer immigrant status should be positively correlated with language skills. Considering further first-stage diagnostic tests, the heteroscedasticity-robust Kleibergen-Paap rank LM statistics^11^ reject the *H*_*0*_ of underidentification in all three versions of instruments. This reinforces the evidence that the instruments are relevant and are correlated with the endogenous regressor of host-country language skills. However, the Hausman specification test does not reject the consistency of OLS under *H*_*0*_ in case of all three sets of IVs, which implies that the differences between the OLS and IV coefficients are not systematic. This latter result suggests that the potential endogeneity bias in OLS may be small in our application.

If we assume a cardinal measure of language skills (columns 5–8), the OLS, IV1 and IVHS coefficient estimates for the local language are all statistically significant, while the same coefficient under IV2 is not. Both the IV1 and IV2 coefficients are smaller than their OLS counterpart, whereas the coefficient under the IVHS approach is virtually equal to the OLS estimate. As regards English skills, the coefficient is positive, statistically significant and broadly similar in size in all four specifications. The first-stage coefficients of IV1 and IV2 take the expected positive sign and are statistically significant. The Kleibergen-Paap rank LM tests reject the null of underidentification in all three cases, meaning that the instruments are relevant and are correlated with the endogenous regressor. However, the Hausman specification test mostly does not reject the validity of OLS under the null hypothesis in columns 6 and 7, but the test rejects the null for the approach in the last column.

Based on Table 8 we can conclude that the IV estimates of the effects of language skills are broadly similar to OLS, when they are statistically significant. This suggests that the endogeneity bias of OLS results seems not too severe. The IV estimates, however, should be handled with some caution. This is because the exclusion restrictions, which cannot be tested directly, may be still violated. In other words, language proximity and age at immigration may also affect income directly, i.e., not only via language skills. In addition, age at immigration, or years of immigrant status may induce a direct learning-by-doing effect on income.

As additional subsample analysis, we replicated the above IV results for the four occupational groups considered previously and for non-EU immigrants. Tables 9 and 10 below report our findings for medium-skilled white-collar and high-skilled occupations. In case of MSWC jobs, the OLS and IVHS estimates for the host-country language are both statistically significant and take similar values, but only when binary language skill indicators are assumed. While the coefficients for English skills are positive and statistically significant in all specifications. These findings are broadly in line with the previous results in Table 6, where language skills were treated as multicategorical variables.

**Table 9:** Returns to language skills of immigrants in medium-skilled white-collar occupations in WE7 countries (OLS vs. IV)

MSWC occupations	(1)	(2)	(3)	(4)	(5)	(6)	(7)	(8)
	OLS	IV1	IV2	IVHS	OLS	IV1	IV2	IVHS
Host-country language prof. (Dummy: ≥ level 3)	0.445* (0.242)	− 0.286 (2.117)	0.147 (3.808)	0.422* (0.251)				
Host-country language prof. (cardinal measure)					0.148 (0.123)	0.048 (0.207)	0.086 (0.288)	0.120 (0.135)
English proficiency (Dummy: ≥ level 3)	0.747*** (0.237)	0.778*** (0.272)	0.760** (0.308)	0.748*** (0.230)				
English proficiency (cardinal measure)					0.305*** (0.091)	0.293*** (0.089)	0.297*** (0.093)	0.301*** (0.088)
First-stage coefficient of IV		0.071***	0.041			0.741***	0.591***	
Kleibergen-Paap rank LM underidentification test (χ^2^)		13.84***	1.56	41.38***		60.88***	33.32***	88.61***
Hausman test χ^2^, OLS vs. IV		0.54	0.02	0.29		2.25	0.31	29.34*
Observations	390	390	390	390	390	390	390	390

Source: Authors’ calculations based on the AES 2016 and ASJP data International weights are used

Robust standard errors are reported in parentheses

***p < 0.01, **p < 0.05, *p < 0.1. WE7 countries: AT, BE, CH, DE, FR, LU, NL. Socio-demographic controls and country dummies were included. In columns IV1, IV2 and IVHS, host-country language proficiency is instrumented by: language proximity (IV1), language proximity interacted with age at immigration (IV2), and the heteroscedasticity-based instruments of Lewbel (2012) (IVHS), respectively

**Table 10:** Returns to language skills of high-skilled immigrants in WE7 countries (OLS vs. IV)

High-skilledoccupations	(1)	(2)	(3)	(4)	(5)	(6)	(7)	(8)
	OLS	IV1	IV2	IVHS	OLS	IV1	IV2	IVHS
Host-countrylanguageprof. (Dummy: ≥ level3)	0.158 (0.284)	3.397** (1.581)	3.860 (2.518)	0.031 (0.308)				
Host-countrylanguageprof. (cardinalmeasure)					0.150* (0.085)	0.302** (0.120)	0.300* (0.170)	0.140 (0.104)
Englishproficiency (Dummy: ≥ level3)	0.168 (0.164)	0.129 (0.171)	0.123 (0.171)	0.170 (0.162)				
Englishproficiency (cardinalmeasure)					0.067 (0.059)	0.070 (0.059)	0.070 (0.059)	0.067 (0.058)
First-stagecoefficientofIV		0.063***	0.047***			0.707***	0.606***	
Kleibergen-PaaprankLMunderidentificationtest(χ^2^)		20.14***	9.71***	107.47***		143.82***	85.65***	119.65***
Hausmantestχ^2^,OLSvs.IV		16.90	8.54	3.19		13.18	4.71	8.79
Observations	1209	1209	1209	1209	1209	1209	1209	1209

Source: Authors’ calculations based on the AES 2016 and ASJP data

International weights are used. Robust standard errors are reported in parentheses

***p < 0.01, **p < 0.05, *p < 0.1. WE7 countries: AT, BE, CH, DE, FR, LU, NL. Socio-demographic controls and country dummies were included. In columns IV1, IV2 and IVHS, host-country language proficiency is instrumented by: language proximity (IV1), language proximity interacted with age at immigration (IV2), and the heteroscedasticity-based instruments of Lewbel (2012) (IVHS), respectively

As regards high-skilled occupations, some of the IV1 and IV2 estimates for host-country language skills in Table 10 are positive and statistically significant, while the OLS coefficient is statistically significant only under the cardinality assumption on the language skills indicators. In either of the cases, the IV1 or IV2 estimates are considerably larger compared to OLS, which may indicate a more substantial endogeneity bias for the subsample of high-skilled occupations. Turning to the coefficients on English skills, they are not statistically significant in any of the specifications for high-skilled jobs in Table 10. This result supports previous findings in Table 6, where multicategorical language skills were assumed.

As for the remaining occupational groups of low-skilled and medium-skilled blue-collar jobs, none of the IV coefficient estimates for language skills were found statistically significant, so the results are moved to the Appendix (Tables 14 and 15). At least in case of low-skilled occupations, the statistical insignificance of the results may be related to low sample size.

In the remaining part of the analysis we looked at IV estimates for the subsample of non-EU immigrants (Table 11). The returns to host-country language skills for this subgroup are somewhat lower than for the full sample when binary language skills indicators are assumed, while the returns are somewhat higher than in the full sample when using cardinal language skills variables. The returns for English are similar to the estimates from the full sample. These findings, especially those for cardinal language skills, are consistent with the results of Table 5 (columns 4 vs. 5), where language skills were measured on a multicategorical scale. In case of binary language skills, however, the results for non-EU immigrants and the full sample (Tables 8 and 11, columns 1–4) are not fully comparable to Table 5 (columns 4–5). This follows from the fact that evidence of income premia to host-country language skills is found for skill levels four and five in Table 5, while the binary indicator of high language skills used in columns 1–4 of Tables 8 and 11 assumes skill level 3 as the cutoff.

**Table 11:** Returns to language skills of immigrants from non-EU countries in WE7 (OLS vs. IV)

Non-EU immigrants	(1)	(2)	(3)	(4)	(5)	(6)	(7)	(8)
	OLS	IV1	IV2	IVHS	OLS	IV1	IV2	IVHS
Host-country language prof. (Dummy: ≥ level 3)	0.354*** (0.120)	1.063* (0.607)	− 1.398 (2.267)	0.379*** (0.143)				
Host-country language prof. (cardinal measure)					0.197*** (0.046)	0.178** (0.075)	− 0.007 (0.122)	0.197*** (0.056)
English proficiency (Dummy: ≥ level 3)	0.398*** (0.140)	0.395*** (0.139)	0.406*** (0.151)	0.398*** (0.139)				
English proficiency					0.169*** (0.044)	0.168*** (0.044)	0.152*** (0.046)	0.169*** (0.044)
(cardinal measure)								
First-stage coefficient of IV		0.092***	0.029**			0.773***	0.526***	
Kleibergen-Paap rank LM underidentification test (χ^2^)		67.32***	4.48**	272.28***		224.09***	114.84***	325.01***
Hausman test χ^2^, OLS vs. IV		3.40	1.49	0.27		0.30	10.34	41.23***
Observations	1693	1693	1693	1693	1693	1693	1693	1693

Source: Authors’ calculations based on the AES 2016 and ASJP data

International weights are used. Robust standard errors are reported in parentheses

***p < 0.01, **p < 0.05, *p < 0.1. WE7 countries: AT, BE, CH, DE, FR, LU, NL. Socio-demographic controls and country dummies were included. In columns IV1, IV2 and IVHS, host-country language proficiency is instrumented by: language proximity (IV1), language proximity interacted with age at immigration (IV2), and the heteroscedasticity-based instruments of Lewbel (2012) (IVHS), respectively

## Conclusion

This paper contributes to the literature on estimating returns to language skills of immigrants with new evidence from 29 European countries. Using the last available wave of Eurostat's Adult Education Survey (2016), we utilize scarce information on immigrants' proficiency levels in multiple languages. We consider immigrants' proficiency in both the local language of the host country and in English, similarly to Lang and Siniver (2009). In contrast to the conclusions of the above authors, however, we find that including English proficiency in the income equation does affect the estimated returns to fluency in the local language. In particular, the estimated returns increase as a result. This suggests the correlation of English proficiency with general unobserved skills of immigrants and, if ignored, may lead to biased estimates.

Further, we show that differentiating the levels of language proficiency matters for estimating income premia, while the common practice in prior research was to use binary indicators. Our estimates suggest that only an almost-native level of fluency in the host country's language yields significantly higher income to immigrants. Whereas in case of English, any additional level of proficiency has a positive effect on income.

Looking at heterogeneity across occupations with different skill requirements, our results indicate evidence of an occupational sorting effect. This occurs when more proficient speakers tend to get more skill-demanding and therefore better paid jobs, which could lead to the overestimation of returns to language proficiency. In accordance with the sorting effect, we show that if we control for skill levels of occupations, the estimated returns to language skills drop. This result is in line with findings in previous literature (e.g., Berman et al. 2003; Boyd and Cao 2009; and McManus et al. 1983). Estimating the returns to language skills for the subsamples of low-skilled, medium-skilled blue-collar, medium-skilled white-collar and high-skilled occupations we find significantly positive returns only for medium-skilled jobs. Focusing on medium skills, English proficiency of immigrants is an asset in both blue- and white-collar jobs, while fluency in the local language is rewarded only in blue-collar professions.

Earlier literature dealt with the potential endogeneity of language skills in the earnings equation. We also attempt to correct our estimates for potential biases due to attenuation, unobserved skills or simultaneity using instrumental variables. First, we use linguistic proximity of the local language and of English to the immigrant's native language and a proxy for age at immigration as instruments for language skills (following Isphording 2013; Clarke and Isphording 2017; and Ghio et al. 2023). Second, we construct instruments identified by heteroskedasticity following the approach of Lewbel (2012). Our first-stage diagnostic tests suggest that all these instruments are relevant and are correlated with the endogenous explanatory variable of host-country language skills. However, the resulting IV estimates are close to the OLS results.

Our findings could be relevant for immigration policies in Europe. The positive labor market outcomes of acquiring proficiency in the local language of the host country by immigrants have been studied extensively in the literature. We add to this stream by new evidence from 29 European countries. Further, in an increasingly globalized world and progressing European economic integration, the role of foreign language skills has become an essential part of human capital. As Isphording (2013) also points out in case of Spain, possible short-term skill gaps in foreign language proficiency in European labor markets may be filled in by immigrants. Accordingly, immigration policies should consider the comparative advantages of immigrants, as well as the skill demands of certain occupations. As our results suggest, medium-skilled jobs tend to reward language skills of immigrants the most.

## Appendix

See Tables 12, 13, 14, 15.

**Table 12:** Manual language code changes for IV estimation

Language/modification	AES code	ASJP code
Albanian	alb	als
Arabic → Cairo Arabic	ara	arz
Aramaic	arc	cld
Armenian	arm	hye
Basque	baq	eus
Cree → Plains Cree	cre	crk
Tlacoatzintepec Chinantec → Chinantec Comaltepec	ctl	cco
Czech	cze	ces
Dutch	dut	nld
Estonian	est	ekk
French	fre	fra
Georgian	geo	kat
German	ger	deu
Ancient Greek → Modern Greek	grc	ell
Modern Greek	gre	ell
Guarani	grn	gug
Chinese → Amoy Minnan/Chaoyang/Dongshan/Hainan Minnan	chi	nan
Icelandic	ice	isl
Kurdish → Kurdish Kurmanji/Northern Kurdish	kur	kmr
Latvia	lav	lvs
Macedonian	mac	mkd
Khalkha Mongolian/Mongolian	mon	khk
Nepali	nep	npi
Persian	per	pes
Pashto, Pushto → Northern Pashto	pus	pbu
Quechua → Atalla/Quechua Abancay Tintay/Quechua Ayacucho	que	quy
Romanian	rum	ron
Slovak	slo	slk
Serbian → Serbocroatian	srp	hbs
Swahili	swa	swh
Classical Syriac → Syrian Arabic	syc	apc
Tibetan	tib	bod
Twi → Twi Asante/Fante	twi	aka
Uzbek → Uzbek	uzb	uzn
Tamazight	zgh	tmz

SIL’s ISO 639 Code Tables (https://iso639-3.sil.org/code_tables/639/data) were used to identify languages in the AES data

**Table 13:** Returns to language skills of immigrants in WE7 countries (Ordered logit estimates)

	(1)	(2)	(3)	(4)	(5)	(6)
	Fulltime and parttime					Fulltime
Years of residence	1.155*** (0.0299)	1.135*** (0.0305)	1.127*** (0.0299)	1.139*** (0.0307)	1.130*** (0.0301)	1.079** (0.0392)
Born outside the EU	0.365*** (0.0515)	0.376*** (0.0537)	0.376*** (0.0539)	0.390*** (0.0561)	0.393*** (0.0566)	0.478*** (0.0819)
Host-country language proficiency		1.221*** (0.0725)		1.272*** (0.0746)		
Host-country lang. prof. (level2)			1.200 (0.321)		1.240 (0.338)	1.160 (0.439)
Host-country lang. prof. (level3)			1.335 (0.371)		1.364 (0.387)	1.248 (0.480)
Host-country lang. prof. (level4)			2.265*** (0.590)		2.319*** (0.612)	1.635 (0.594)
Host-country lang. prof. (level5)			2.080*** (0.554)		2.731*** (0.796)	1.972* (0.743)
English proficiency				1.320*** (0.0715)		
English proficiency (level1)					1.728** (0.427)	1.611 (0.528)
English proficiency (level2)					2.214*** (0.640)	2.420** (0.919)
English proficiency (level3)					2.972*** (0.707)	3.139*** (1.032)
English proficiency (level4)					3.269*** (0.979)	4.366*** (1.711)
English proficiency (level5)					4.182*** (1.644)	3.284** (1.810)
Socio-demographic controls	Yes	Yes	Yes	Yes	Yes	Yes
Country dummies	Yes	Yes	Yes	Yes	Yes	Yes
Observations	3700	3700	3700	3700	3700	2524

Source: Authors’ calculations based on the AES 2016 data. Note: International weights are used. Odds ratios are reported. Robust standard errors are reported in parentheses. ****p* < 0.01, ***p* < 0.05, **p* < 0.1. WE7 countries: AT, BE, CH, DE, FR, LU, NL. Socio-demographic controls include: education attainment level, part-time dummy, potential experience, degree of urbanisation, marital status, birthplace and citizenship

**Table 14:** Returns to language skills of low-skilled immigrants in WE7 countries (OLS vs. IV)

Low-skilledoccupations	(1)	(2)	(3)	(4)	(5)	(6)	(7)	(8)
	OLS	IV1	IV2	IVHS	OLS	IV1	IV2	IVHS
Host-country language prof. (Dummy: ≥ level3)	0.382 (0.276)	− 0.204 (1.265)	− 1.793 (3.502)	0.382 (0.260)				
Host-country language prof. (cardinal measure)					0.048 (0.121)	− 0.059 (0.187)	− 0.302 (0.357)	0.048 (0.114)
English proficiency (Dummy: ≥ level3)	0.148 (0.310)	− 0.008 (0.448)	− 0.432 (0.974)	0.148 (0.291)				
English proficiency (cardinal measure)					− 0.025 (0.101)	− 0.059 (0.109)	− 0.138 (0.142)	− 0.025 (0.095)
First-stage coefficient of IV		0.107**	0.048			0.760***	0.419**	
Kleibergen-Paap rank LM underidentification test (χ^2^)		5.67**	0.87			22.97***	6.59**	
Hausman test χ^2^,OLS vs. IV		0.40	0.59	0.00		0.94	1.74	6.12
Observations	201	201	201	201	201	201	201	201

Source: Authors’ calculations based on the AES 2016 and ASJP data. International weights are used. Robust standard errors are reported in parentheses. ****p* < 0.01, ***p* < 0.05, **p* < 0.1. WE7 countries: AT, BE, CH, DE, FR, LU, NL. Socio-demographic controls and country dummies were included. In columns IV1, IV2 and IVHS, host-country language proficiency is instrumented by: language proximity (IV1), language proximity interacted with age at immigration (IV2), and the heteroscedasticity-based instruments of Lewbel (2012) (IVHS), respectively

**Table 15:** Returns to language skills of immigrants in medium-skilled blue-collar occupations in WE7 countries (OLS vs. IV)

MSBCoccupations	(1)	(2)	(3)	(4)	(5)	(6)	(7)	(8)
	OLS	IV1	IV2	IVHS	OLS	IV1	IV2	IVHS
Host-country language prof. (Dummy: ≥ level3)	0.201(0.160)	0.650(1.310)	− 1.328(2.886)	0.171(0.181)				
Host-country language prof. (cardinal measure)					0.119*(0.069)	0.106(0.109)	− 0.016(0.155)	0.048(0.114)
English proficiency (Dummy: ≥ level3)	0.193(0.277)	0.207(0.271)	0.143(0.298)	0.192(0.272)				
English proficiency (cardinal measure)					0.128(0.085)	0.124(0.087)	0.090(0.097)	− 0.025(0.095)
First-stage coefficient of IV		0.067***	0.033**			0.814***	0.615***	
Kleibergen-Paap rank LM underidentification test (χ^2^)		27.84***	4.69**	101.02***		105.82***	57.71***	164.46***
Hausman test χ^2^,OLS vs. IV		0.34	0.74	0.13		0.08	2.96	6.95
Observations	719	719	719	719	719	719	719	719

Source: Authors’ calculations based on the AES 2016 and ASJP data. International weights are used. Robust standard errors are reported in parentheses. ****p* < 0.01, ***p* < 0.05, **p* < 0.1. WE7 countries: AT, BE, CH, DE, FR, LU, NL. Socio-demographic controls and country dummies were included. In columns IV1, IV2 and IVHS, host-country language proficiency is instrumented by: language proximity (IV1), language proximity interacted with age at immigration (IV2), and the heteroscedasticity-based instruments of Lewbel (2012) (IVHS), respectively

## Footnotes

1 The instruments are a dummy variable indicating whether the individual got married overseas, number and age of children and the birthplace concentration variable.

2 The authors use age at arrival to the US interacted with a dummy for non-English-speaking country as the identifying instrument. A similar instrument based on children’s age at immigration was used on Australian data by Budría and Martínez-de-Ibarreta (2021).

3 The authors use three binary instruments: an indicator whether the respondent arrived in Spain before the age of 12, an indicator whether the respondent has a child who is proficient in Spanish and an indicator of the respondent’s willingness to stay in Spain for the next five years.

4 Note that more detailed information on the structure of earnings in the household is not available in the AES dataset.

5 The classification of foreign language skill levels used in the survey is based on the Council of Europe scale. Foreign language skills are determined based on the respondents’ own assessment of their skill level.

6 https://asjp.clld.org/software.

7 We consider skill levels three and above to represent high skills.

8 Note that the homoscedasticity of U is testable only partially (see Pagan and Hall 1983), as the rejection of the null hypothesis may be the consequence of a heteroscedastic *V*_*1*_. In contrast, the heteroscedasticity of *V*_*2*_ can be tested by the standard Breusch and Pagan (1979) test.

9 See Sect. 3 for a detailed list of countries included.

10 See Sect. 3 for further details.

11 See Kleibergen and Paap (2006).
